# Sequential Fermentation with Selected Immobilized Non-*Saccharomyces* Yeast for Reduction of Ethanol Content in Wine

**DOI:** 10.3389/fmicb.2016.00278

**Published:** 2016-03-11

**Authors:** Laura Canonico, Francesca Comitini, Lucia Oro, Maurizio Ciani

**Affiliations:** Dipartimento Scienze della Vita e dell'Ambiente, Università Politecnica delle MarcheAncona, Italy

**Keywords:** ethanol reduction, immobilized cells, non-*Saccharomyces* yeast, sequential fermentation, wine

## Abstract

The average ethanol content of wine has increased over the last two decades. This increase was due to consumer preference, and also to climate change that resulted in increased grape maturity at harvest. In the present study, to reduce ethanol content in wine, a microbiological approach was investigated, using immobilized selected strains of non-*Saccharomyces* yeasts namely *Starmerella bombicola, Metschnikowia pulcherrima, Hanseniaspora osmophila*, and *Hanseniaspora uvarum* to start fermentation, followed by inoculation of free *Saccharomyces cerevisiae* cells. The immobilization procedures, determining high reaction rates, led a feasible sequential inoculation management avoiding possible contamination under actual winemaking. Under these conditions, the immobilized cells metabolized almost 50% of the sugar in 3 days, while *S. cerevisiae* inoculation completed all of fermentation. The *S. bombicola* and *M. pulcherrima* initial fermentations showed the best reductions in the final ethanol content (1.6 and 1.4% v/v, respectively). Resulting wines did not have any negative fermentation products with the exception of *H. uvarum* sequential fermentation that showed significant amount of ethyl acetate. On the other hand, there were increases in desirable compounds such as glycerol and succinic acid for *S. bombicola*, geraniol for *M. pulcherrima* and isoamyl acetate and isoamyl alcohol for *H. osmophila* sequential fermentations. The overall results indicated that a promising ethanol reduction could be obtained using sequential fermentation of immobilized selected non-*Saccharomyces* strains. In this way, a suitable timing of second inoculation and an enhancement of analytical profile of wine were obtained.

## Introduction

Over the last few decades, there has been a progressive increase in the ethanol content in wine due to new wine styles arising from consumer preference, and to the global climate change that is often associated with increased grape maturity (Jones et al., 2005; Grant, 2010; MacAvoy, 2010; Alstona et al., 2011; Gonzalez et al., 2013). However, wine with high levels of ethanol can be perceived negatively due to health concerns, wine quality reduction and taxation rates according to ethanol content (Guth and Sies, 2001; Athès et al., 2004; Gawel et al., 2007).

In this context, several lines of research are aimed at reducing the ethanol content of wines, which have generally focused on vineyard management and winemaking practices, and particularly on the de-alcoholization of wine (Belisario-Sánchez et al., 2009; Kutyna et al., 2010; Stoll et al., 2010; Schmidtke et al., 2012; Bindon et al., 2013). Considering microbiological applications, several strategies that use genetically modified *Saccharomyces cerevisiae* strain have also been proposed for reduction of alcohol content in wine (Ehsani et al., 2009; Kutyna et al., 2010; Varela et al., 2012). More recently, Tilloy et al. (2014) used evolution-based strategies together with breeding strategies to show that evolved or hybrid strains can led to ethanol reductions of 0.6 to 1.3% (v/v) in comparison with the ancestral strains.

Another approach to reduce the production of ethanol might be the use of non-*Saccharomyces* wine yeast as part of the natural microbiota present on grapes and winemaking equipment during grape juice fermentation (Renouf et al., 2005, 2007). The use of non-*Saccharomyces* yeast in combination with *S. cerevisiae* has been proposed to improve the quality and enhance the complexity of wine (Jolly et al., 2014; Capozzi et al., 2015). Thus, the use of controlled multistarter fermentation using selected cultures of non-*Saccharomyces* and *S. cerevisiae* yeast strains has been encouraged (Ciani and Comitini, 2011; Comitini et al., 2011; Domizio et al., 2011; Magyar and Tóth, 2011; Di Maio et al., 2012; Ehsani et al., 2012; Morata et al., 2012; Jolly et al., 2014). In this context, non-*Saccharomyces* wine yeast and multistarter fermentation might have a role in the reduction of the ethanol content in wine. The wide variability amongst non-*Saccharomyces* yeast regarding ethanol yield, fermentation efficiency, biomass production, by-product formation, and respiro-fermentative metabolism might be used to reduce the ethanol concentration in wine. Among non-*Saccharomyces* wine yeasts some strains/species showed low ethanol yield and sugar consumption by respiration (Crabtree negative). Using these selected strains, 1–2% v/v of ethanol reduction was achieved but prolonged time of sequential inoculation or high level of acetic acid were shown (Contreras et al., 2014; Gobbi et al., 2014; Quirós et al., 2014).

Sequential fermentation adequately setup might be an attractive tool for the use of non-*Saccharomyces* yeast for the reduction of the ethanol content in wine. This fermentative approach, in which an initial inoculation of a non-*Saccharomyces* strain is followed by inoculation of the *S. cerevisiae* starter strain, would allow the metabolism of the first inoculated yeast to be exploited without too great an influence on the *S. cerevisiae* strain. To benefit from the metabolic particularities of some non-*Saccharomyces* yeast in sequential fermentation (i.e., low ethanol yield, low fermentation efficiency), the inoculation level and the duration of the interval between the first and second inoculations are fundamental. An enhancement of the inoculation level of non-*Saccharomyces* yeast will improve the competitiveness toward wild yeast and *S. cerevisiae* starter strain and, at the same time, this will increase the expression of their metabolic activity.

In the present study, we evaluated the initial use of immobilized non-*Saccharomyces* yeast in sequential fermentation trials in terms of reduction of the ethanol content in the wine. The immobilization procedures allowed high inoculation rates, with the consequent high reaction rates, to reduce the delay before the *S. cerevisiae* starter strain inoculation. This also avoids possible contamination under actual winemaking conditions, due to this late inoculation of *S. cerevisiae*.

## Materials and methods

### Yeast strains

The non-*Saccharomyces* yeast strains used in this study were *Starmerella bombicola* (formerly named *Candida stellata*) DiSVA66, (DBVPG # 3827; Industrial Yeast Collection of the University of Perugia), *Metschnikowia pulcherrima* DiSVA269, *Hanseniaspora osmophila* DiSVA253, and *Hanseniaspora uvarum* DiSVA252. These were obtained from the Yeast Collection of the Department of Life and Environmental Sciences (DiSVA) of the Polytechnic University of Marche (Italy). All of the strains were previously selected and used in mixed fermentation trials to enhance the analytical and aromatic profile of the wine, as well as to improve the wine complexity (Ciani and Ferraro, 1998; Comitini et al., 2011; Domizio et al., 2011). These were used here as the initial fermentation trials for sequential fermentations with *S. cerevisiae* commercial strain Lalvin EC1118 (Lallemand Inc., Toulouse, France), which was also used in pure culture as the control.

All of the strains were maintained at −80°C for long-term storage, in cryovials supplemented with 80% (w/v) glycerol as the cryoprotective agent. Subsequently, the strains were cultured on Yeast Peptone Dextrose (YPD) agar medium at 25°C for 48–72 h, and stored at 4°C.

## Media

### Synthetic grape juice

Synthetic grape juice (SGJ) for the micro-fermentation trials was prepared using three different solutions: solution A (500 mL), solution B (250 mL), and solution C (250 mL). The three solutions were sterilized at 121°C for 20 min separately and then combined aseptically (Ciani and Ferraro, 1996). Solution A contained 110 g D-glucose, 110 g D-fructose, 10 mg ergosterol, and 1 ml Tween 80. Four milliliters of ergosterol stock solution (Tween 80, 6.25 mL; ergosterol, 62.5 mg in ethanol to make 25 mL) was added to the glucose-fructose solution to complete solution A. Solution B contained 6 g L-(+)-tartaric acid, 3 g L-(−)-malic acid, and 0.5 g citric acid. Solution C was a mix of 1.7 g Yeast Nitrogen Base without amino acids and without ammonium sulfate (DIFCO), 0.2 g CaCl_2_, 2 g casamino acids, 0.8 g arginine-HCl, 1 g L-(2)-proline, and 0.1 g L-(2)-tryptophan. Solutions B and C were buffered at pH 3.5 with NH_4_OH and H_3_PO_4_, respectively.

### Natural grape juice

Natural grape juice (NGJ) was obtained during 2014 vintage and came from Verdicchio, a white grape variety that is grown in the Marche region, in central Italy. The main characteristics of the grape juice were: pH 3.39; total acidity, 8.27 g/L; free SO_2_, 12 mg/L; total SO_2_, 48 mg/L; malic acid, 3.3 g/L; initial sugar content, 202 g/L; yeast assimilable nitrogen, 160 mg N/L.

### Immobilization procedures

Modified YPD medium (0.5% yeast extract, 0.1% peptone, 2% dextrose, all w/v) was used to produce biomass at 25°C for 72 h in a rotary shaker (150 rpm). This biomass, for the immobilization system, were harvested by centrifugation, washed three times with sterile distilled water and added to 2.5% Na-alginate (Carlo Erba, Milan, Italy), at a ratio of 5% (wet w/v). Using a peristaltic pump, this mixture was then dripped into CaCl_2_ (0.1 M) to induce gelation. After 1 h, the beads formed were washed several times with sterile distilled water and then used immediately. The inoculum for the immobilized cells of the non-*Saccharomyces* was 10% (wet w/v), which corresponded to an inoculum of *ca*. 2 × 10^8^ cells/mL in SGJ or NGJ.

### Fermentation conditions

To evaluate the influence of the sequential inoculations using immobilized non-*Saccharomyces* cells on the ethanol content, several fermentation trials were set up with SGJ and NGJ. In SGJ, the inoculated immobilized non-*Saccharomyces* cells were removed after 48 or 72 h, and the free *S. cerevisiae* cells (1 × 10^6^ cell/mL) were inoculated into the partially fermented grape juice. In NGJ, the sequential fermentation trials were conducted by inoculation of the *S. cerevisiae* starter culture after only 72 h, with and without the removal of the immobilized cells.

Cultures of *S. cerevisiae* were pre-incubated in SGJ at 25°C in a rotary shaker (150 rpm) for 48 h, harvested by centrifugation, and washed with sterile distilled water, with the procedure standardized to provide an inoculation level of 1 × 10^6^ cells/mL. Before the *S. cerevisiae* inoculation, 1 g of beads containing non-*Saccharomyces* yeast were collected and maintained under agitation in 50 mL 1% Na-citrate solution (w/v) for 1 h, to release the cells. Cell viability was then evaluated by standard plate counting techniques, in YPD medium. Parallel control trials were carried out using free *S. cerevisiae* cells only.

The fermentation trials were carried out in 1-L glass minifermenters that contained 400 mL SGJ or NGJ under static conditions at 25°C and in duplicate. The minifermenters had two ports, one for gas flow and the other for the inoculation of beads, and a septum of a glass frit, to maintain the beads in the medium and to allow carbon dioxide to come out.

The weight loss of the minifermenters due to CO_2_ evolution was followed until the end of the fermentation trials (constant weight for three consecutive days). Samples of media and beads were taken after 48, 72 h, and at the end of the fermentation and underwent chemical and microbiological analysis respectively.

### Analytical determinations

Ethanol was measured by gas-liquid chromatography (GLC) analysis (AOAC, 1990). Acetaldehyde, ethyl acetate, and higher alcohols were determined by direct injection into the GLC system. Samples were injected into a column of 30 m × 0.32 mm, with 0.25 μm film thickness (Zebron ZB-WAXPlus; Phenomenex, Torrance, California, USA) with an internal standard of 1-pentanol (162 mg/L). Nitrogen was used as the carrier gas. A Shimadzu gas chromatograph (Japan) equipped with a flame ionization detector was used. The oven temperature ranged from 40°C to 200°C. The temperature of the injector and the detector was 220°C. The volatile compounds were extracted using an ether–hexane (1:1) extraction technique, and evaluated by capillary GLC. For quantification, and before their extraction, the samples were spiked with a known amount of 3-octanol, as the internal standard (1.6 mg/l). A glass capillary column was used: 0.25 μm Supelcowax 10 (length, 60 m; internal diameter, 0.32 mm). One microliter was injected in split–splitless mode, with 60 s splitless; temperature of injection, 220° C; temperature of detector, 250°C; carrier gas, helium; and flow rate, 2.5 mL/min. The temperature program was: 50°C for 5 min; 3°C /min to 220°C, and then 220°C for 20 min. The compounds were identified and quantified by comparisons with external calibration curves for each compound. The glucose, fructose, glycerol, and succinic acid concentrations were determined using specific enzyme kits (Megazyme International Ireland). Volatile acidity (expressed as grams acetic acid per liter) was quantified by steam distillation, according to the official analytical methods (EC, 2000).

### Statistical analysis

Analysis of variance (ANOVA) was applied to the experimental data for the main enological characteristics of the wines. The means were analyzed using the STATISTICA 7 software. The significant differences were determined using Duncan tests, and the data were considered significant if the associated *P*-values were < 0.05.

## Results

### Fermentation kinetics and main fermentation parameters of synthetic grape juice trials

**Figure 1:**
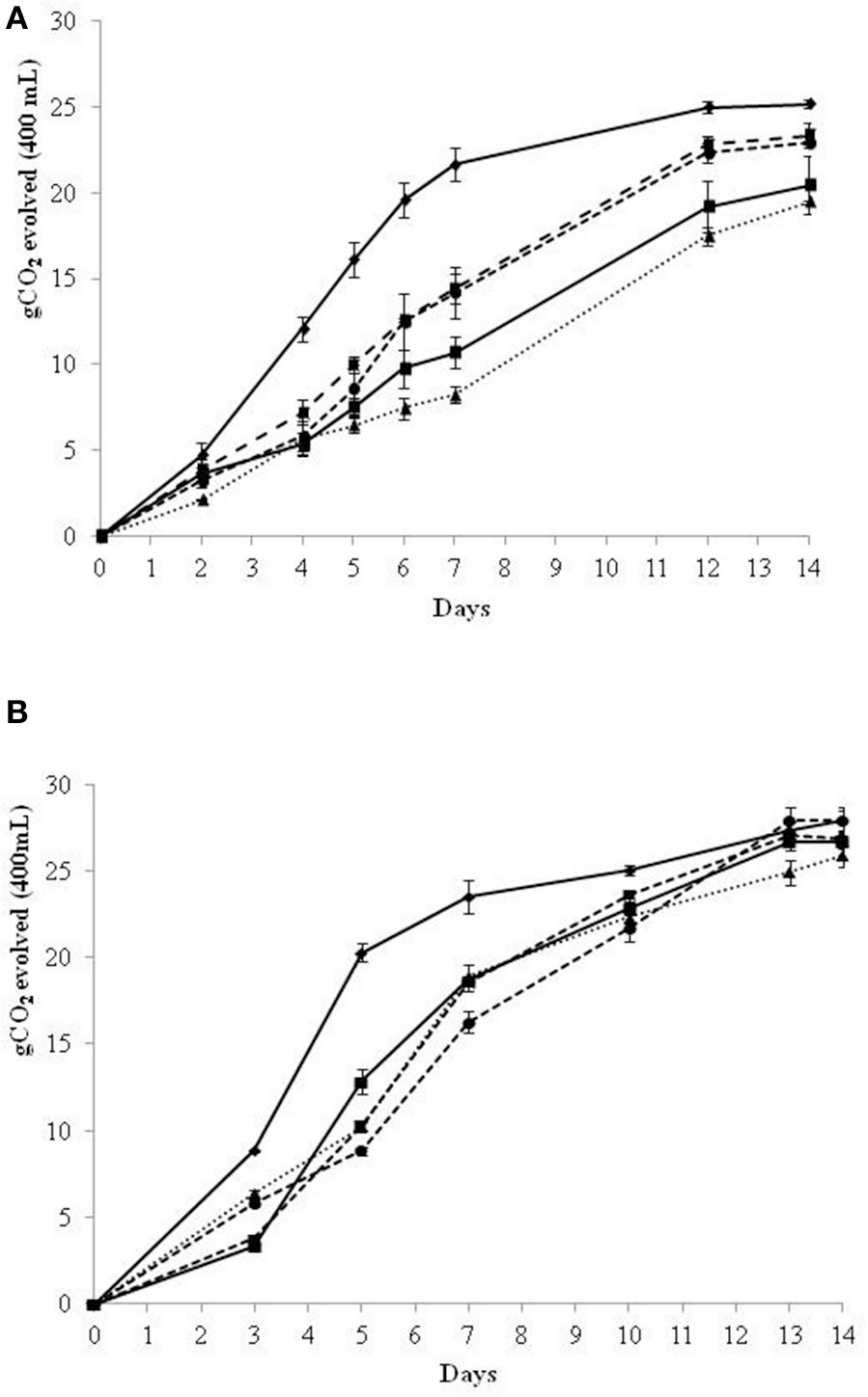
**Growth kinetics in sequential fermentation trials of immobilized non-*Saccharomyces* yeast and control *S. cerevisiae* on synthetic grape juice (SGJ)**. Beads were removed at 48 h **(A)** and 72 h **(B)** of fermentation. 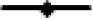
*S. cerevisiae* control culture; 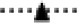
*H. osmophila/S. cerevisiae*; 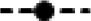
*S. bombicola/S. cerevisiae*; 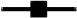 *H. uvarum/S. cerevisiae*; 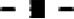 *M. pulcherrima/S. cerevisiae*.

In the SGJ fermentation trials, the inoculated immobilized non-*Saccharomyces* cells were removed after 48 and 72 h, and the partial fermented SGJ was then inoculated with free *S. cerevisiae* cells (Figure 1). The 48 h SGJ trials (Figure 1A) showed that the control *S. cerevisiae* improved the fermentation kinetics compared to the sequential fermentation trials with the initial non-*Saccharomyces* yeast. The sequential fermentation trials in SGJ with *M. pulcherrima* and *S. bombicola* showed overlapping fermentation kinetics that were quicker than for the *H. uvarum* and *H. osmophila*. Moreover, all of the sequential fermentation trials showed less final CO_2_ evolved when compared to the control *S. cerevisiae*.

The data for the fermentation parameters of the sequential fermentations inoculated after 48 h in SGJ are summarized in Table 1. All of the sequential fermentation trials showed significantly lower ethanol content when compared with the control *S. cerevisiae*, with residual sugar less than 3 g/L. In particular, the sequential fermentation trials carried out with *M. pulcherrima, S. bombicola*, and *H. uvarum* showed comparable lower final ethanol production, while with *H. osmophila* there was higher ethanol content. Data for the ethanol yield basically confirmed those of ethanol production. At 48 h of fermentation, the control *S. cerevisiae* showed a sugar consumption of 25%, while the non-*Saccharomyces* sequential fermentation trials using the immobilized cells, showed significantly lower sugar consumption, from 11 to 21%; with the exception of *M. pulcherrima* (38%). Again, and with the exception of *M. pulcherrima*, the other sequential fermentation trials resulted in significant increases in glycerol, in comparison with the control *S. cerevisiae*. For the volatile acidity, there were no significant differences seen, while the succinic acid content significant increased in all fermentation trials (with the exception of *H. osmophila*) with the immobilized non-*Saccharomyces* yeast, particularly with *S. bombicola*.

**Table 1:** **The main fermentation parameters for non-*Saccharomyces* yeast in sequential fermentation trials with synthetic grape juice, with *S. cerevisiae* EC1118 inoculated after 48 h of fermentation**.

**Fermentation trials**	**Sugar consumed at 48 h (%)**	**End of fermentation**					
		**Sugar consumed (g/L)**	**Ethanol (% v/v)**	**Ethanol yield (g/g)**	**Glycerol (g/L)**	**Volatile acidity (as acetic acid g/L)**	**Succinic acid (g/l)**
*S. cerevisiae* control culture	24.56 ± 0.62^a^	218.77 ± 0.10^b^	12.54 ± 0.02^a^	0.452 ± 0.001^a^	5.32 ± 0.44^b^	0.38 ± 0.02^d^	0.37 ± 0.04^c^
*H. osmophila/S. cerevisiae*	20.98 ± 0.01^b^	217.83 ± 0.05^c^	11.76 ± 0.12^b^	0.426 ± 0.004^b^	6.70 ± 0.02^a^	0.54 ± 0.01^c^	0.44 ± 0.06^b, c^
*M. pulcherrima/S. cerevisiae*	38.33 ± 0.01^c^	219.41 ± 0.04^a^	11.40 ± 0.07^c^	0.410 ± 0.002^c^	5.64 ± 0.29^b^	0.63 ± 0.02^b^	0.53 ± 0.04^b^
*S. bombicola/S. cerevisiae*	17.42 ± 0.75^d^	217.67 ± 0.14^c^	11.36 ± 0.01^c^	0.412 ± 0.000^c^	7.54 ± 0.70^a^	0.57 ± 0.00^c^	1.03 ± 0.01^a^
*H. uvarum/S. cerevisiae*	10.56 ± 1.71^e^	218.83 ± 0.05^b^	11.48 ± 0.00^c^	0.414 ± 0.000^c^	6.99 ± 0.07^a^	0.68 ± 0.01^a^	0.49 ± 0.01^b^

The initial sugar concentration was 220 g/L. Data are means ± standard deviations from two independent experiments. Data with different superscript letters (

a, b, c, d, e *) within each column are different according to Duncan tests (0.05%)*.

Since, 48 h sequential fermentation showed a limited sugar consumption and with the aim to enhance the fermentation performance of immobilized cells, 72 h sequential fermentation was evaluated. In 72 h SGJ sequential fermentation trials (Figure 1B), the control *S. cerevisiae* showed essentially the same fermentation kinetics as described above for the 48 h trials. Over these first 3 days of fermentation with the immobilized non-*Saccharomyces* species, *H. osmophila*, and *S. bombicola* showed enhanced fermentation kinetics in comparison with *M. pulcherrima* and *H. uvarum*. After removal of the beads and inoculation of *S. cerevisiae*, all of the sequential fermentation trials showed lower fermentation kinetics when compared with the control *S. cerevisiae*, although all condition achieved comparable amounts of CO_2_ evolution at the end of the fermentation trials.

In this regard, the inoculation delay for the addition of free *S. cerevisiae* cells from 48 to 72 h resulted in further reductions in ethanol content in comparison with the control *S. cerevisiae*, paired with a small increase in the residual sugars (Table 2). *H. uvarum* was the only sequential fermentation trial that did not show any difference in ethanol reduction from the 48 to 72 h inoculation of *S. cerevisiae*. Data for the ethanol yield confirmed this trend. Indeed, and with the exception of *H. uvarum*, all of these sequential fermentation trials showed a significant reduction in ethanol yield in comparison with the control *S. cerevisiae*. As expected, there was increased sugar consumption of the immobilized non-*Saccharomyces* yeast from 48 to 72 h, which varied from 37 to 52% of total sugars. For glycerol content, only the *S. bombicola* and *H. uvarum* sequential fermentation trials showed a significant increase when compared with the control *S. cerevisiae* (Table 2). All sequential fermentation trials showed significant, but limited, increases in volatile acidity and succinic acid (although the succinic acid content did not reach statistical significance for *H. uvarum*).

**Table 2:** **The main fermentation parameters for non-*Saccharomyces* yeast in sequential fermentation trials with synthetic grape juice, with *S. cerevisiae* EC1118 inoculated after 72 h of fermentation**.

**Fermentation trials**	**Sugar consumed at 72 h (%)**	**End of fermentation**					
		**Sugar consumed (g/L)**	**Ethanol (% v/v)**	**Ethanol yield (g/g)**	**Glycerol (g/L)**	**Volatile acidity (as acetic acid g/L)**	**Succinic acid (g/L)**
*S. cerevisiae* control culture	43.82 ± 0.03^b^	220.00 ± 0.11^a^	12.36 ± 0.27^a^	0.443 ± 0.009^a^	5.18 ± 0.07^c^	0.36 ± 0.00^d^	0.39 ± 0.00^c^
*H. osmophila/S. cerevisiae*	52.16 ± 2.30^a^	216.90 ± 0.28^c^	11.03 ± 0.16^b^	0.401 ± 0.005^b, c^	5.25 ± 0.17^c^	0.66 ± 0.01^a^	0.71 ± 0.10^b^
*M. pulcherrima/S. cerevisiae*	37.22 ± 2.12^d^	217.79 ± 0.04^b^	11.01 ± 0.22^b^	0.400 ± 0.008^c^	5.07 ± 0.02^c^	0.51 ± 0.01^b^	0.59 ± 0.03^b^
*S. bombicola/S. cerevisiae*	42.20 ± 1.63^b^	216.74 ± 0.14^c^	11.08 ± 0.00^b^	0.403 ± 0.000^b, c^	7.46 ± 0.01^a^	0.49 ± 0.01^b, c^	1.12 ± 0.10^a^
*H. uvarum/S. cerevisiae*	39.54 ± 071^c^	215.86 ± 0.37^d^	11.58 ± 0.43^b^	0.423 ± 0.000^a, b^	6.18 ± 0.19^b^	0.47 ± 0.01^c^	0.55 ± 0.01^b, c^

The initial sugar concentration was 220 g/L. Data are means ± standard deviations of two independent experiments. Data with different superscript letters (

a, b, c, d *) within each column are different according to Duncan tests (0.05%)*.

### Fermentation kinetics and main fermentation parameters of natural grape juice trials

**Figure 2:**
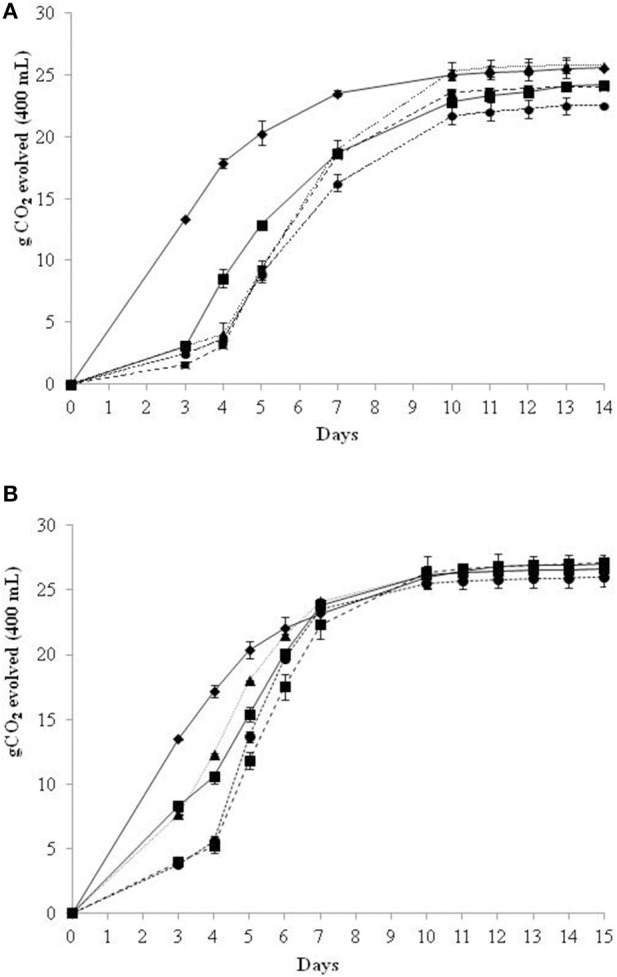
**Growth kinetics in sequential fermentation trials of immobilized non-*Saccharomyces* yeast and control *S. cerevisiae* on natural grape juice (NGJ)**. Beads were removed at 72 h **(A)** and without beads removal **(B)**: 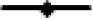
*S. cerevisiae* control culture; 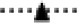 *H. osmophila/S. cerevisiae*;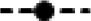 *S. bombicola/S. cerevisiae*; 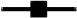 *H. uvarum/S. cerevisiae*; 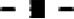 *M. pulcherrima/S. cerevisiae*.

After identifying the delay time of the inoculum of *S. cerevisiae* starter (72 h) that allows the immobilized cells to consume around 50% of initial sugars in a synthetic medium, we carried out non-*Saccharomyces* immobilized cells sequential fermentations in NGJ (Verdicchio grape juice) to evaluate the overall fermentation parameters and the analytical profile of wines. The fermentation kinetics of the sequential fermentation trials in NGJ, conducted with the removal of the immobilized cells, showed similar behaviors to those in SGJ for 72 h (Figure 2A). Different fermentation kinetics were shown in NGJ without the removal of beads, and hence in the continued presence of the immobilized non-*Saccharomyces* species (Figure 2B). These data highlighted that in the presence of the immobilized cells during the whole fermentation process, this resulted in increased fermentation kinetics by day 7, with the same CO_2_ evolved as for the control *S. cerevisiae*. After this time, all of the fermentation kinetics showed overlap (Figure 2B).

The data regarding to the main fermentation parameters in NGJ both with or without beads removal are reported in Table 3, and they confirm the significantly lower ethanol content in all of the sequential fermentation trials, in comparison with the control *S. cerevisiae*, which were all accompanied by little or no residual sugars (< 2 g/L).

**Table 3:** **The main fermentation parameters for non-*Saccharomyces* yeast in sequential fermentation trials with natural grape juice, with *S. cerevisiae* EC1118 inoculated after 72 h of fermentation and without removing the beads**.

**With removing beads**						**Without removing the beads**				
**Fermentation trials**	***S. cerevisiae* control culture**	***H. osmophila/S. cerevisiae***	***M. pulcherrima/S. cerevisiae***	***S. bombicola/S. cerevisiae***	***H. uvarum/S. cerevisiae***	***S. cerevisiae* control culture**	***H. osmophila/S. cerevisiae***	***M. pulcherrima/S. cerevisiae***	***S. bombicola/S. cerevisiae***	***H. uvarum/S. cerevisiae***
Sugar consumed at 72 h (%)	47.72 ± 0.75^b^	54.76 ± 1.94^a^	39.80 ± 0.38^g^	41.34 ± 0.77^e, f^	43.57 ± 2.17^c^	48.46 ± 0.02^b^	55.26 ± 0.26^a^	40.79 ± 0.19^f, g^	42.08 ± 0.003^d, e^	42.83 ± 0.72^c, d^
Sugar consumed (g/L)	201.39 ± 0.14^a^	199.74 ± 0.03^c^	199.74 ± 0.03^c^	199.67 ± 0.13^c^	200.83 ± 0.0^b^	200.58 ± 0.46^b^	200.81 ± 0.31^b^	200.62 ± 0.38^b^	201.04 ± 0.25^a, b^	200.90 ± 0.2^a, b^
Ethanol (% v/v)	12.06 ± 0.04^a^	11.12 ± 0.11^c^	11.06 ± 0.09^c, d^	10.99 ± 0.06^d^	11.12 ± 0.04^c^	12.00 ± 0.03^a^	11.00 ± 0.04^d^	10.54 ± 0.03^f^	10.36 ± 0.05^g^	10.79 ± 0.05^e^
Ethanol yield (g/g)	0.476 ± 0.001^a^	0.439 ± 0.004^c^	0.437 ± 0.003^c, d^	0.434 ± 0.002^d, e^	0.436 ± 0.00^c, d^	0.472 ± 0.001^b^	0.432 ± 0.002^e^	0.414 ± 0.002^g^	0.406 ± 0.002^h^	0.423 ± 0.002^f^
Glycerol (g/L)	5.87 ± 0.19^d, e^	5.36 ± 0.08^f^	5.88 ± 0.11d^e^	7.63 ± 0.04^b^	6.02 ± 0.03^d^	5.68 ± 0.27^e^	4.80 ± 0.18^g^	5.80 ± 0.30^d, e^	8.43 ± 0.20^a^	6.74 ± 0.21^c^
Volatile acidity (as acetic acid g/L)	0.56 ± 0.01^c, d^	0.49 ± 0.0^d^	0.58 ± 0.02^b, c^	0.55 ± 0.1^c, d^	0.67 ± 0.00^a^	0.63 ± 0.07^b, c^	0.59 ± 0.03^a, b, c^	0.65 ± 0.04^a, b^	0.58 ± 0.02^b, c^	0.67 ± 0.06^a^
Succinic acid (g/L)	0.52 ± 0.02^d, e^	0.70 ± 0.10^c^	0.60 ± 0.02^c^	0.90 ± 0.04^b^	0.47 ± 0.00^e^	0.53 ± 0.01^d, e^	0.65 ± 0.02^*c, d*^	0.66 ± 0.11^c, d^	1.16 ± 0.03^a^	0.57 ± 0.12^c, d, e^

The initial sugar concentration was 202 g/L. Data are means ± standard deviations. Data with different superscript letters (

a, b, c, d, e, f, g *) within each column are different according to Duncan tests (0.05%)*.

The same was also observed for the ethanol yield, which confirmed that the reduction of ethanol content was mainly due to the lower yield. In this context, the main by-products showed some variations: the *S. bombicola* sequential fermentation trial confirmed the highest production of glycerol and succinic acid as previously reported (Ciani and Ferraro, 1998), while *H. osmophila* showed the lowest glycerol content. Compared to the control *S. cerevisiae*, in the NGJ sequential trials *H. uvarum* showed a significant increase in volatile acidity, while the other sequential fermentation trials showed comparable or lower values.

### Viability and cell release from the beads

Two important features to monitor in the use of these immobilized cells are the loss of cell viability and the cells released from the beads, which is closely related to the conservation of the structure of the matrix. The data reported in Table 4 show that in SGJ, the viability of all of the non-*Saccharomyces* yeast after 48 and 72 h was around 1 × 10^9^ cell/g, without significant loss of cell viability after their use. The low levels of cells released after their use (*ca*. 1 × 10^2^ cell/mL after 48 h, and 1 × 10^3^ cell/mL after 72 h) confirmed the high cell viabilities, thus indicating the good integrity of the beads. In NGJ, there was comparable high cell viability, although there was also an increase in the cell release (about 1 × 10^4^ cell/mL), which indicated some break-up of the matrix.

**Table 4:** **Non-*Saccharomyces* viable cell counts and cells released from the beads in synthetic grape juice and natural grape juice at 48 and 72 h of fermentation trials**.

**Fermentation trials**	**Synthetic grape juice**				**Natural grape juice**	
	**48 h**		**72 h**		**72 h**	
	**Viable cells (Log cell/g)**	**Cells released (Log CFU/mL)**	**Viable cells (Log cell/g)**	**Cells released (Log CFU/mL)**	**Viable cells (Log cell/g)**	**Cells released (Log CFU/mL)**
*H. osmophila/S. cerevisiae*	9.64 ± 0.01	2.28 ± 0.04	9.64 ± 0.02	3.30 ± 0.05	9.36 ± 0.21	4.19 ± 0. 20
*M. pulcherrima/S. cerevisiae*	9.58 ± 0.82	2.03 ± 0.01	9.40 ± 0.02	3.02 ± 0.03	8.60 ± 0.34	4.64 ± 0.11
*S. bombicola/S. cerevisiae*	9.40 ± 0.70	2.57 ± 0.04	9.58 ± 0.40	3.56 ± 0.03	8.60 ± 0.34	4.60 ± 0.06
*H. uvarum/S. cerevisiae*	9.36 ± 0.24	2.02 ± 0.01	9.36 ± 0.03	3.25 ± 0.10	8.82 ± 0.21	4.03 ± 0.01

### Ethanol reduction

**Figure 3:**
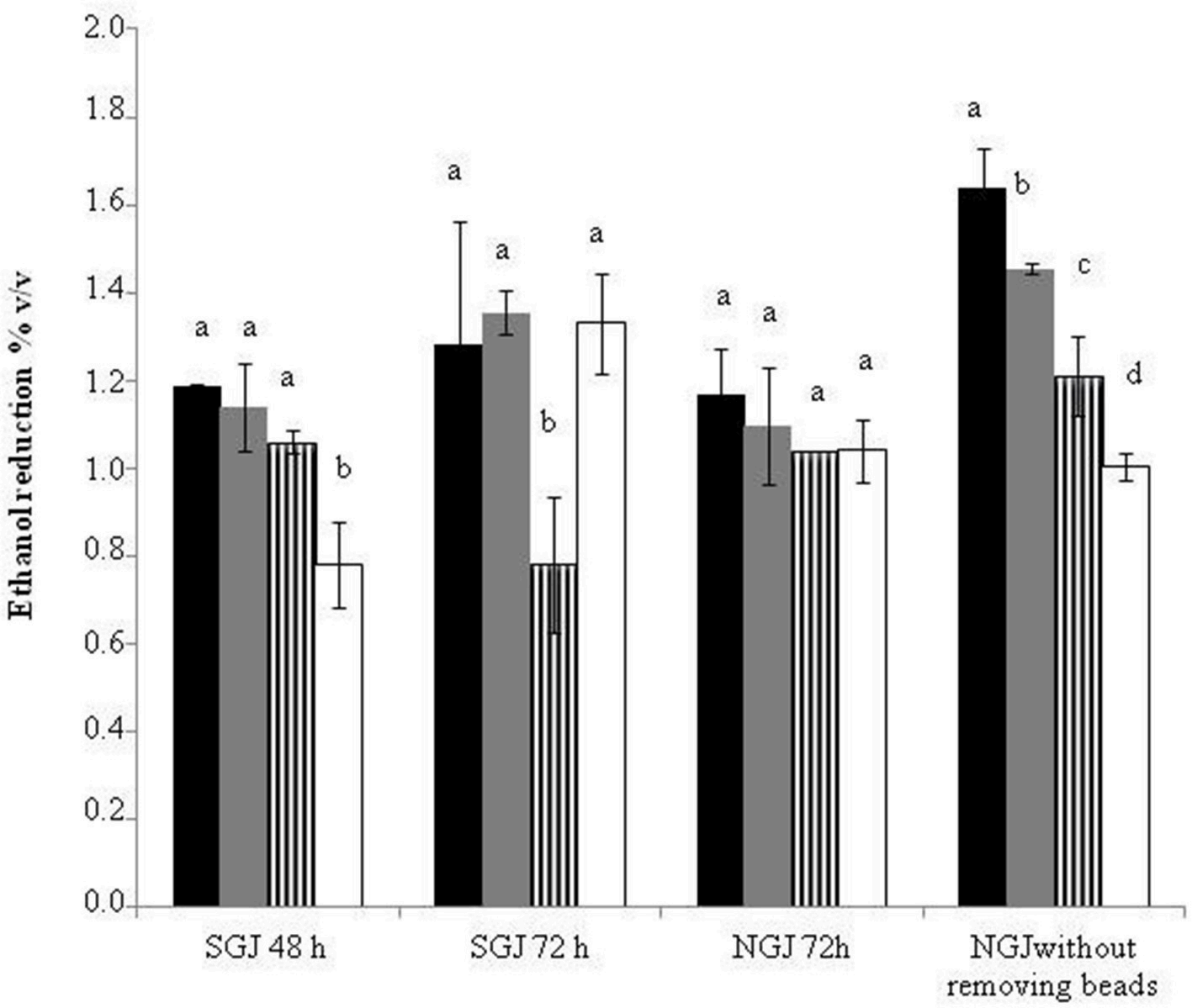
**Ethanol reduction with synthetic grape juice and natural grape juice**. 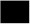 *S. bombicola/S. cerevisiae*; 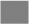 *M. pulcherrima/S. cerevisiae*; 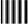 *H. uvarum/S. cerevisiae*; 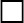 *H. osmophila/S. cerevisiae*. Data with different superscript letters (^a, b, c, d^) within each trials are different according to Duncan tests (0.05%).

Figure 3 summarizes the ethanol reductions obtained across all of the sequential and control fermentation trials. The data for SGJ indicate that the delay in the *S. cerevisiae* inoculation from 48 to 72 h generally promoted further ethanol reductions. Indeed, from 48 to 72 h, the ethanol reductions were from 1.18 to 1.28% (v/v) for *S. bombicola*, from 1.14 to 1.35% (v/v) for *M. pulcherrima*, and from 0.78 to 1.33% (v/v) for *H. osmophila*. In contrast, this was opposite for *H. uvarum*, that ranged from 1.06 to 0.78% (v/v). In NGJ, the immobilized non-*Saccharomyces* yeast showed a comparable or little bit lower ethanol reduction than that exhibited by sequential fermentation trials at 72 h in SGJ, without any significant differences among the non-*Saccharomyces* species. However, significantly greater reductions in ethanol were obtained in the trials without the beads removal, thus leaving the non-*Saccharomyces* yeast in the fermentation trials with the *S. cerevisiae*. In these fermentation trials, there were generally significant improvements in the ethanol reductions over the 72 h NGJ sequential fermentation trials: from 1.10 to 1.46% (v/v) for *M*. *pulcherrima*, from 1.17 to 1.64% (v/v) for *S. bombicola*, and from 1.04 to 1.21% (v/v) for *H. uvarum*. Only the *H. osmophila* sequential fermentation trial did not show any statistically significant variation here (from 1.04 to 1.00% [v/v]).

### The main volatile compounds in sequential fermentations on natural grape juice

To determine the influence of the non-*Saccharomyces* sequential fermentation trials on the aromatic profile of the wine, the main volatile compounds on NGJ were assayed, and the results are summarized in Table 5.

**Table 5:** **The main volatile compounds (mg/L) of sequential fermentation trials in natural grape juice with *S. cerevisiae* EC1118 inoculated after 72 h of fermentation with and without removing beads**.

**With removing beads**						**Without removing beads**				
**Fermentation trials**	***S. cerevisiae* control culture**	***H. osmophila/S. cerevisiae***	***M. pulcherrima/S. cerevisiae***	***S. bombicola/S. cerevisiae***	***H. uvarum/S. cerevisiae***	***S. cerevisiae* control culture**	***H. osmophila/S. cerevisiae***	***M. pulcherrima/S. cerevisiae***	***S. bombicola/S. cerevisiae***	***H. uvarum/S. cerevisiae***
**ESTERS**										
Ethyl butyrate	0.17 ± 0.01^e^	0.20 ± 0.01^c^	0.21 ± 0.01^c^	0.25 ± 0.04^b^	0.20 ± 0.01^c^	0.19 ± 0.01^e^	0.20 ± 0.06^c^	0.21 ± 0.00^d^	0.26 ± 0.01^b^	0.53 ± 0.02^a^
Ethyl acetate	36.94 ± 0.69^f^	51.52 ± 0.03^e^	83.10 ± 2.12^c^	36.68 ± 1.64^f^	195.79 ± 2.9^a^	36.94 ± 0.40^f^	68.04 ± 3.35^d^	87.42 ± 0.51^c^	45.44 ± 0.31^e^	165.68 ± 0.9^b^
Phenyl ethylacetate	0.043 ± 0.007^a^	0.036 ± 0.004^a^	0.037 ± 0.004^a^	0.027 ± 0.001^a^	0.035 ± 0.01^a^	0.031 ± 0.002^a^	0.029 ± 0.014^a^	0.032 ± 0.007^a^	0.027 ± 0.007^a^	0.034 ± 0.00^a^
Ethyl hexanoate	0.11 ± 0.01^a^	0.09 ± 0.00^b, c^	0.10 ± 0.00^a, b^	0.01 ± 0.00^f^	0.08 ± 0.01^c^	0.11 ± 0.001^a^	0.05 ± 0.03^d^	0.03 ± 0.00^e^	0.01 ± 0.00^f^	0.11 ± 0.00^a^
Ethyl octanoate	0.089 ± 0.003^a, b^	0.020 ± 0.00^e^	0.084 ± 0.001^a, b^	0.096 ± 0.00^a^	0.032 ± 0.01^d, e^	0.076 ± 0.002^b^	0.028 ± 0.037^d, e^	0.074 ± 0.001^b^	0.059 ± 0.00^c^	0.037 ± 0.00^d^
Isoamyl acetate	0.20 ± 0.01^d^	0.27 ± 0.10^c^	0.22 ± 0.01^d^	0.22 ± 0.01^d^	0.22 ± 0.01^d^	0.22 ± 0.06^d^	0.41 ± 0.02^a^	0.41 ± 0.01^a^	0.33 ± 0.02^b^	0.22 ± 0.01^d^
**ALCOHOLS**										
n-propanol	64.2 ± 0.95^d^	35.7 ± 0.4^e^	36.3 ± 0.3^e^	86.5 ± 0.4^b^	34.5 ± 2.7^e^	65.1 ± 0.5^d^	75.1 ± 1.5^c^	106.0 ± 2.3^a^	57.7 ± 1.8^d^	88.1 ± 2.4^b^
Isobutanol	8.6 ± 1.1^b^	7.0 ± 1.1^b^	4.4 ± 0.1^b, c^	4.5 ± 0.7b^c^	4.2 ± 1.7^b, c^	7.9 ± 2.8^b^	5.6 ± 2.2^b, c^	4.0 ± 0.8b^c^	5.2 ± 0.2^b, c^	11.6 ± 3.4^a^
Amylc alcohol	10.2 ± 0.3^b, c^	8.6 ± 0.5^b, c^	15.7 ± 1.2^a^	8.6 ± 1.4^b, c^	10.6 ± 2.4^b^	9.3 ± 0.02^b, c^	16.1 ± 0.1^a^	10.1 ± 2.7^b, c^	11.1 ± 0.4^b^	10.2 ± 2.7^b, c^
Isoamylic alcohol	99.8 ± 0.1^c^	106.8 ± 0.4^b^	94.9 ± 0.7^c, d^	81.9 ± 0.8^e^	84.7 ± 2.4^e^	97.9 ± 1.2^c^	129.4 ± 0.2^a^	106.6 ± 1.9^b^	92.1 ± 0.1^c, d^	83.7 ± 0.1^e^
β-Phenyl ethanol	30.8 ± 1.2^a, b^	23.3 ± 0.5^c, d^	21.0 ± 1.4^d^	26.9 ± 0.7^b, c^	23.3 ± 0.5^c, d^	31.9 ± 2.9^a, b^	19.8 ± 2.1^d^	32.6 ± 1.7^a^	30.2 ± 1.6^a, b^	20.3 ± 3.1^d^
Hexanol	0.047 ± 0.000^a, b^	0.049 ± 0.001^a^	0.026 ± 0.00^e^	0.040 ± 0.003^c^	0.27 ± 0.00^e^	0.048 ± 0.001^a, b^	0.048 ± 0.02^a^	0.035 ± 0.004^d^	0.047 ± 0.00^a, b^	0.043 ± 0.004 ^b, c^
**CARBONYL COMPOUNDS**										
Acetaldehyde	53.3 ± 0.7^f^	113.3 ± 1.4^b^	116.9 ± 1.4^b^	87.14 ± 3.4^d^	112.3 ± 3.1^b^	51.3 ± 0.4^f^	95.2 ± 0.8^c^	73.8 ± 2.6^e^	70.1 ± 2.7^e^	126.2 ± 2.9^a^
Acetoin	ND	ND	ND	ND	ND	ND	ND	ND	ND	ND
**MONOTERPENS**										
Linalool	0.006 ± 0.001^a^	0.005 ± 0.002^a, b^	0.005 ± 0.00^a, b^	0.003 ± 0.00^b^	0.003 ± 0.00^b^	0.006 ± 0.00^a^	0.006 ± 0.00^a^	0.004 ± 0.001^a, b^	0.005 ± 0.00^a, b^	0.003 ± 0.00^b^
Nerol	0.217 ± 0.002^a^	0.081 ± 0.003^c^	0.065 ± 0.001^d^	0.048 ± 0.003^e^	0.030 ± 0.003^f^	0.216 ± 0.002^a^	0.074 ± 0.035^c, d^	0.077 ± 0.004^c^	0.096 ± 0.007^b^	0.063 ± 0.00^d^
Geraniol	0.188 ± 0.012^b^	0.193 ± 0.010^b^	0.266 ± 0.002^a^	0.153 ± 0.001^c^	0.111 ± 0.01^e^	0.193 ± 0.010^b^	0.131 ± 0.097^d^	0.255 ± 0.015^a^	0.138 ± 0.006^c, d^	0.149 ± 0.00^c^

Data are means ± standard deviations of two independent experiment. Data with different superscript letters (

a, b, c, d, e, f ) within each column are different according to Duncan tests (0.05%). ND = not detected

An increase in ethyl acetate was showed in all sequential fermentations with the exception of *S. bombicola* trials, in both with or without beads removal. However, only *H. uvarum* sequential fermentation showed unacceptable level of this compound (around the threshold value for a negative impact on the aromatic profiles amounting to 175 mg/L).

Regarding to the other esters, all sequential fermentations showed comparable or lower amount of phenyl ethyl acetate, ethyl hexanoate, and ethyl octanoate when compared with the control *S. cerevisiae* while significant increases in ethyl butyrate content was showed in *S. bombicola* (both with and without beads removal trials) and *H. uvarum* (only without beads removal). Furthermore, *H. osmophila* sequential fermentation showed an increase in isoamyl acetate, which is responsible of the fruity note, in both trials while *M. pulcherrima* and *S. bombicola* sequential fermentations exhibited an enhancement of this ester only without beads removal trials.

Regarding to the higher alcohols, sequential fermentations showed variable production of n-propanol. Isobutanol, amylic alcohol, and hexanol did not show significant differences between sequential fermentations and control trials while an increase in isoamyl alcohol in *H. osmophila* and *M. pulcherrima* but only without beads removal trials was found. A generalized reduction in β-phenyl ethanol content was shown in all sequential fermentation trials when compared with the control *S. cerevisiae*. On the contrary, an enhancement in acetaldehyde content was found in all sequential fermentations.

Regarding the volatile terpenes, a significant increase in geraniol content was exhibited by *M. pulcherrima* sequential fermentation confirming the capability of this yeast strain to liberate volatile terpens by glycosidase activity (Comitini et al., 2011).

## Discussion

Different microbiological approaches have been proposed to reduce the ethanol content in wine, such as genetically modified *S. cerevisiae* yeast (Ehsani et al., 2009; Kutyna et al., 2010; Varela et al., 2012; Rossouw et al., 2013), evolution-based strategies, together with breeding strategies (Abalos et al., 2011; Tilloy et al., 2014) and the use of non-*Saccharomyces* wine yeast (Contreras et al., 2014; Gobbi et al., 2014; Quirós et al., 2014).

In this last approach, the strategies include the need to manage the fermentation on the basis of several enological traits of the non-*Saccharomyces* species used. Several wine yeast species could be selected for their low ethanol yield, alcoholic fermentation efficiency, biomass and by-product formation as a result of the diversion of carbon away from ethanol production (Ciani and Maccarelli, 1998; Gobbi et al., 2014). On the other hand, these non-*Saccharomyces* wine yeasts could be used to promote sugar consumption via respiration rather than fermentation, through partial aeration of the grape juice (Gonzalez et al., 2013).

Both these approaches have indicated the promising use of non-*Saccharomyces* wine yeast to limit ethanol production. The use of various amounts of oxygen added during the first stages of fermentation of mixed fermentations results in significant reduction in ethanol production (Contreras et al., 2015b; Morales et al., 2015). Indeed, under limited aerated conditions, *M. pulcherrima, Torulaspora delbrueckii*, and *Zygosaccharomyces bailii* mixed fermentations resulted in reduced ethanol content, from 1.5 to 2.2% (v/v), while under high agitation and aeration rates this resulted in unacceptable amounts of acetic acid by *S. cerevisiae* partner strain (Quirós et al., 2014; Contreras et al., 2015b). However, the effects of aerobic conditions on the analytical and sensorial profiles and oxygen modulation in mixed fermentations were not evaluated and for these reasons, the aeration of grape juice requires further investigations. Recently, under anaerobic conditions, a reduction in the alcohol level was achieved in fermentations performed using sequential inoculation with a strain of *M. pulcherrima* (Contreras et al., 2014, 2015a). Using a strain of *M. pulcherrima* in sequential fermentation trials, 50% of sugar consumed was achieved in white and red grape juice, with a delay of the second inoculation with *S. cerevisiae* strain of 9 and 17 days resulting in an ethanol reduction of 0.9 and 1.6% (v/v), respectively (Contreras et al., 2014). However, a long delay for timing of second inoculation is difficult to manage under winery conditions, because of wild microflora contamination, where the competitiveness of the non-*Saccharomyces* strain is low, and the wild *S. cerevisiae* strains can easily dominate the fermentation process.

In this context, the management of non-*Saccharomyces* yeast in mixed fermentation trials with the aim to reduce the ethanol content is a crucial step. Indeed, to achieve sugar consumption of about 50% using non-*Saccharomyces* yeast as the starter culture, a long delay of the *S. cerevisiae* starter inoculation would be needed. In the present study, we evaluated the use of four selected non-*Saccharomyces* strains in immobilized forms in both SGJ and NGJ, to obtain high inoculation levels and the consequent high metabolic activity, to reduce the time of the second inoculation. Under these conditions, with a delay of 3 days, we obtained a sugar consumption that ranged from 40 to 54% with an ethanol reduction from 1.0 to 1.17% (v/v). Without removing beads, the alcohol reduction was further enhanced. Under these conditions, *M. pulcherrima* and *S. bombicola* confirmed the benefits for ethanol reduction in mixed fermentation trials than for those with *H. uvarum* and *H. osmophila*, which showed alcohol reductions of 1.4 and 1.6% v(/v), respectively. As previously shown, *M. pulcherrima* and *S. bombicola* in mixed fermentations, can reduce the ethanol content (Ciani and Ferraro, 1998; Contreras et al., 2014, 2015a,b; Quirós et al., 2014). The significant enhancement of by-products such as glycerol or succinic acid do not justify the ethanol reduction obtained. Other fermentation by-products that were not evaluated in this study and coming from glycerol-pyruvic fermentation or other metabolic pathways could explain this result. Moreover, pyruvate-metabolism is strictly linked to amino-acids, organic acids, and lipids biosynthesis and, consequently, sugar carbon could follow these pathways (Gancedo and Serrano, 1989).

Another important feature that should be highlighted is the analytical profile of final wines. In this context, the wine obtained showed in general comparable or better analytical profiles then for the control *S. cerevisiae*. Indeed, all fermentation trials showed a limited increase of acetaldehyde content and only *H. uvarum* sequential fermentation exhibited significant amount of ethyl acetate that negatively affect the aroma profile of final wine. On the other hand, an enhancement in sequential fermentations of some desired compounds was shown. In particular, *S. bombicola* showed an enhancement in of glycerol and succinic acid, *M. pulcherrima* exhibited an increase in geraniol while *H. osmophila* displayed a significant increase in isoamyl acetate and isoamyl alcohol.

In conclusion, the non-*Saccharomyces M. pulcherrima* and *S. bombicola* are both promising wine yeast species for use in immobilized forms in sequential fermentation trials to reduce the ethanol content in wine. The use of high inoculation levels and immobilization procedures, however, results in substantial increases in the management costs of the fermentation process. For these reasons, further investigations are necessary to explore reductions in the bead concentrations, modulation of grape juice aeration, and evaluation of the sensorial profile of the resulting wine.

## Funding

The work was financially supported by Ricerca Scientifica di Ateneo RSA2014 and RSA2015.

## Author contributions

LC, LO, FC, and MC contributed equally to this manuscript. All authors participated in the design and discussion of the research. LC and LO carried out the experimental part of the work.LC, LO, MC, and FC carried out the analysis of the data and wrote the manuscript. All authors have read and approved the final manuscript.

### Conflict of interest statement

The authors declare that the research was conducted in the absence of any commercial or financial relationships that could be construed as a potential conflict of interest.
